# Conducting operational research in humanitarian settings: is there a shared path for humanitarians, national public health authorities and academics?

**DOI:** 10.1186/s13031-020-00280-2

**Published:** 2020-05-13

**Authors:** Enrica Leresche, Claudia Truppa, Christophe Martin, Ariana Marnicio, Rodolfo Rossi, Carla Zmeter, Hilda Harb, Randa Sami Hamadeh, Jennifer Leaning

**Affiliations:** 1 International Committee of the Red Cross Delegation, Beirut, Lebanon; 2 Harvard TH Chan School of Public Health, Boston, USA; 3 International Committee of the Red Cross, Geneva, Switzerland; 4 Lebanese Ministry of Public Health, Beirut, Lebanon; 5 Harvard François Xavier Bagnoud Center for Health and Human Rights, Boston, USA

**Keywords:** Humanitarian response, Operational research, Research partnership, Protracted crisis, Evidence-based humanitarian action, Co-production

## Abstract

In humanitarian contexts, it is a difficult and multi-faceted task to enlist academics, humanitarian actors and health authorities in a collaborative research effort. The lack of research in such settings has been widely described in the past decade, but few have analysed the challenges in building strong and balanced research partnerships. The major issues include considering operational priorities, ethical imperatives and power differentials. This paper analyses in two steps a collaborative empirical endeavour to assess health service utilization by Syrian refugee and Lebanese women undertaken by the International Committee of the Red Cross (ICRC), the Lebanese Ministry of Public Health (MoPH) and the Harvard François-Xavier Bagnoud (FXB) Center.

First, based on challenges documented in the literature, we shed light on how we negotiated appropriate research questions, methodologies, bias analyses, resource availability, population specificities, security, logistics, funding, ethical issues and organizational cultures throughout the partnership.

Second, we describe how the negotiations required each partner to go outside their comfort zones. For the academics, the drivers to engage included the intellectual value of the collaboration, the readiness of the operational partners to conduct an empirical investigation and the possibility that such work might lead to a better understanding in public health terms of how the response met population needs. For actors responding to the humanitarian crisis (the ICRC and the MOPH), participating in a technical collaboration permitted methodological issues to be worked through in the context of deliberations within the wider epistemic community.

We find that when they collaborate, academics, humanitarian actors and health authorities deploy their respective complementarities to build a more comprehensive approach. Barriers such as the lack of uptake of research results or weak links to the existing literature were overcome by giving space to define research questions and develop a longer-term collaboration involving individual and institutional learning. There is the need ahead of time to create balanced decision-making mechanisms, allow for relative financial autonomy, and define organizational responsibilities. Ultimately, mutual respect, trust and the recognition of each other’s expertise formed the basis of an initiative that served to better understand populations affected by conflict and meet their needs.

## Background

This paper presents a structured analysis of a multi-disciplinary research partnership formed to assess a humanitarian response to a protracted crisis. It aims to address the challenges described in the literature regarding efforts to conduct a collective research process in a humanitarian context. This analysis derives from the experience gained through the collaborative engagement in Lebanon of a humanitarian organization, an academic centre, and a government agency. The research initiative focused on utilization of Primary Health Care (PHC) services by Lebanese and Syrian women and extended for over 4 years, from the inception of the project until the first peer-reviewed publication [1].

### Research gap

Conducting operational research in the context of a humanitarian response is a difficult, challenging but still much needed enterprise [2–5]. Research conducted in humanitarian settings has increased but remains of insufficient quantity and quality [2, 3]. At the same time, demands for greater accountability [2, 3, 6, 7] and questions around equity in the research process are rising [8–11].

In the context of dispersed refugees and Internally Displaced Populations (IDPs), research efforts with strong methodologies are especially scarce [2, 3]. There is a documented disparity between regions, with a gap noted for the Middle East [2, 12] except for specific over-researched communities [13] and, despite key initiatives, a gap also remains on Sexual and Reproductive Health (SRH) [12, 14–17].

A recent series on “health in humanitarian crises” described the need to improve the quantitative and qualitative evidence; to measure health outcomes in terms of mortality and morbidity; and to strengthen research on safe access to facilities and affected populations [2, 4, 6, 7]. Analysis of processes that overcome some of the key challenges are explored in global health research [18–20] but to a much lesser extent in the humanitarian world [15, 21]. Little is known on how to build formal ventures involving humanitarian actors, national stakeholders and academics in a region such as the Middle East, which has been heavily affected by conflict in the past decade [12]. Also under-researched in these settings are negotiations around resource distribution (access to grants, academic expertise, understanding of the global political context, access to the field) [8–10] or around cognitive and moral dynamics (notions of trust, ethical issues, direct ties with communities) [11, 13].

### 10 key documented challenges of conducting research in humanitarian settings

We first conducted a scoping review of the literature in English, including a recent academic series on evidence in humanitarian settings [2–4, 6, 21], research published by humanitarian actors including methodological papers [5, 22–25], academic analyses of humanitarian and global health partnerships [12, 20, 26, 27], research on SRH and conflicts including the Middle East [14–17] as well as literature related to research ethics and in conflict settings [8–11, 13]. Then, we compared issues emerging from the literature with our recent experience and we collectively agreed upon a set of 10 key challenges reflecting the main trade-offs we had to negotiate throughout the partnership. We discussed what important factors where at play while building practical solutions to meet each challenge. We also looked at how the same body of literature described limitations of stand-alone approaches as opposed to a partnership. Finally, we reflected on whether the ways that our partnership had been initiated, built and managed contributed to our meeting these 10 challenges and we sought to define main take-home findings for each partner.

The 10 key challenges documented in the literature are outlined below and will be discussed in-depth in the body of the debate.

1. Using the right methodology based on an appropriate research question constitutes a significant challenge [2, 3, 6, 17, 22–24, 27]
2. Failing to account for bias, study limitations, and lack of statistical data are also seen as major shortcomings [2, 12, 15, 21]
3. Specifying the population to be studied in conflict affected areas (including populations on the move) involves balancing issues of comprehensiveness and practicality [2, 6, 13, 21]
4. Measuring the initial health status of displaced populations is difficult especially in conflicts of long duration where essential baseline information is usually missing [3, 14, 28, 29]
5. Securing the functional balance of resources (such as financial, technical, human, and time) may prove daunting [2, 5, 15, 16, 21–23, 25, 26]
6. Adapting methodologies for field conditions becomes troublesome because lengthy prospective cohort studies or randomized controlled trials (RCTs) are difficult to conduct in unpredictable and volatile environments [2, 21, 23]
7. Constraining research efforts are distortions imposed by issues of security and logistics [2, 3, 5, 11–13, 15–17, 28]
8. Unstable and unpredictable funding patterns restrain the perceived scope of research [2, 3, 21, 23]
9. Ethical issues are complex and in certain situations of marked power differentials can appear prohibitive [2, 5, 6, 8–11, 13, 30]
10. The differences in the analytic cultures of humanitarian as compared to academic actors constitute yet another type of barrier [2, 21–24, 31]

### Research driven by academics

In the past decades, several initiatives have been launched to strengthen global and humanitarian research capacity [2, 3, 7, 15, 18, 26, 32]. Existing academic field collaborations include partnerships between universities, non-governmental organizations (NGOs) or academic networks in relatively stable environments [15, 18, 19, 26]. Recent academic proposals to promote research in the humanitarian field include establishing a global research service linked to existing coordination bodies “probably housed by academic centres of excellence” [3]. Yet processes driven by academics in the global north often retain the main roles of access to funding and control of study design and analysis, while NGO field personnel or national academic partners perform the tasks of community engagement, data collection and initial analysis [8, 9, 18, 33]. These patterns of power and roles may carry the risk that the senior academic managers lack awareness of the relevance of the research question to field operations and that field stakeholders do not contribute substantively to the research question, data analysis and interpretation. It is also possible the implementers fear a diversion of resources from the beneficiaries and do not incorporate results into subsequent programs and that key decision-makers may dismiss the process as a top-down academically driven activity, resulting in a possible weak impact on the program [19, 23, 25, 32, 34]. In unbalanced partnerships, academic institutions may be perceived as owning key ideas and results [8, 18] and thus might miss the contributions of field actors relating to their insights on equity considerations, community engagement, policy making and benefits to the local population [9, 18, 19, 33].

### Research driven by humanitarian actors

In the past decades humanitarian actors such as Médecins Sans Frontières (MSF) have made significant efforts to develop research within the humanitarian sector including funding, training of staff, ethical review processes and engagement in academic debates [22–24, 32, 35, 36]. Global partnerships such as the Structured Operational Research and Training Initiative (SORT IT) have expanded human resource capacities to conduct operational research in humanitarian settings [25, 35, 37]. Between 2009 and 2014, 236 participants were trained over a period of 9 to 12 months resulting in 186 manuscripts published [35]. These efforts grounded in the field, close to beneficiaries and dealing with operationally relevant questions have substantially increased the number of peer reviewed publications [25, 35, 37]. Major issues persist, including the absence of actors from the Middle East involved in such global initiatives [25, 35] and challenges of maintaining a research community in conflict affected regions where access is variable [2, 13, 15]. Other concerns include the perception of research diverting operational funds and resources, the long time for implementation of the study results, the lack of writing skills for publication and the need for substantial mentorship [18, 25, 32]. Research driven by humanitarian actors also may lack the capacity or support to assess results in the context of broader concerns in the existing literature, an assessment that could contribute critical insights on local operations and local findings [18, 19, 21, 22].

## Main text

### Aim

In humanitarian settings, insecurity, lack of social and economic supports and precarious legal status affect the population to be studied. In such contexts research outcomes have important political, financial and operational implications for a multitude of intertwined stakeholders [6, 21, 22]. Health authorities, international actors, and academics have different perspectives, technical capacities, resources, and priorities. It is in the interest of all, however, to ensure that the humanitarian response is appropriate, reaches the most vulnerable, and mitigates the effects of the crisis on the population and the implicated health system [21, 38–40].

The first objective of this paper is to identify retrospectively key factors that allowed the ICRC, the Lebanese Ministry of Public Health (MoPH) and the Harvard FXB Center for Health and Human Rights (FXB Center) to overcome key documented barriers in a partnership to conduct field operational research in Lebanon [1].

The second objective is to explore how each partner was able to bridge the divide between humanitarian or academic driven research. Through the experience of our multi-disciplinary team in the context of Lebanon, we identify a collaboration pathway that required both academics and humanitarians to get out of their comfort zones. We discuss factors at the intersection between and among humanitarians, academics and national health authorities that need to be addressed in order to build a robust research partnership in a protracted crisis setting.

This paper does not consider the implementation of operational changes as a result of this research. We recognize that operational research in humanitarian settings has important ethical and operational dimensions related to its usefulness and implementation, especially once scarce resources have been invested to perform the research [5]. However, the focus of this work is to analyse the joint enterprise itself. The use and implementation of research results will be elaborated in a subsequent paper.

### The three actors

**The ICRC** mandate since 1863 has been to protect the life and dignity of victims of armed conflict and in other situations of violence and provide assistance within the frame of the Geneva Conventions [41]. The ICRC has been present continuously in Lebanon since 1967. In 2015, the ICRC scope of activities changed significantly to support the Lebanese health system response to the needs of an estimated total of up to 1.5 million registered and unregistered Syrian refugees present in the country [38, 42].

**The MoPH** and public healthcare system in Lebanon has long been subjected to political and economic unrest [43, 44]. In the past the MoPH was heavily affected by the Lebanese civil war and since 2011 has been hit hard by the Syrian refugee crisis, resulting in the Lebanese society hosting the highest per capita concentration of refugees in the world [38, 43–45]. Refugees are scattered among the poorest Lebanese in informal tent settlements in rural areas or in overcrowded urban areas including Palestinian camps [38, 46]. Over 50% of the refugees are estimated to be women and children [17, 46, 47]. Syrian refugees are granted access to the same channels of healthcare as Lebanese through a network of PHC services embedded in a complex privatized system [43, 44]. In 2017, Syrian refugees constituted half of the total beneficiaries in the MoPH network [48]. The majority of deliveries among younger women are by Syrians, constituting 70.3% of all deliveries under the age of 20 with a maternal mortality ratio double that among Lebanese in 2016 and 2017 [49]. The refugee demand for public healthcare services in Lebanon has coincided with independent efforts by the MoPH to promote domestic access to the MoPH PHC network [43, 50]. These two combined trends might have strained important aspects of the health system.

**The FXB Center** is an academic institution focusing on research related to provision of health care and other rights-based supports and protections for vulnerable populations in volatile settings. It conducts action-oriented research to support policy and advocacy for the promotion of human rights and adherence to norms of international humanitarian law in contexts of armed conflict, forced migration, and widespread social distress.

### Factors precipitating the partnership

Some important factors had an influence on the creation and subsequent trajectory of the partnership. Two international forums on the need to reach Every Woman and Every Child Everywhere (EWEC) in Abu-Dhabi and in Washington in 2015 nurtured discussions between the FXB Center and the ICRC around issues of measuring access to supported services at population levels [40]. Despite ICRC’s long experience in providing medical assistance to victims of armed conflict, evidence was missing on interventions that improved access measured at the population level, apart from important but relatively isolated efforts to assess quality of care mechanisms [51, 52] or immunization campaigns [53].

For the MoPH, the observed patterns of utilization in reproductive health services raised questions about access and referrals in the existing response to the crisis. The MoPH also saw the increasing demand for health services from the refugees in the context of a progressive funding gap, which increased from 24% (US $29 million) in 2013 to 55% USD ($159 million) in 2018 [54]. These unmet shortfalls forced the public system to absorb the cost—mitigated only marginally by imposition of higher out-of-pocket expenditures for certain services. The accumulated health budget deficit in 2018 (estimated at 15 million USD) led to a gradual increase in poverty for the crisis-affected populations [38], triggering operational questions on how to increase services utilization for affected Lebanese and Syrians women in particular. The heavy burden on the Lebanese public health system, the protracted characteristics of the crisis, combined with the presence of over 100 NGO partners [38] led the ICRC to re-examine its strategy for support.

Discussions with the MoPH brought all three actors in 2016 to work on a concept note as the basis for the research design. The key question became: Was the ICRC primary health care support reaching those most affected by the crisis and matching beneficiaries’ needs in terms of access, cost and appropriateness? The research aim was to evaluate ICRC support in response to the Syrian crisis, to inform evidence-based health programming, and to nurture a set of discussions around policy with the MoPH in Lebanon [1].

### How 10 documented challenges were approached

The section below addresses the influence of access to resources and the management of cognitive and moral dynamics. Resources include financial means, technical skills, contextual understanding, operational experience and access to the field. Dynamics refer to building trust, maintaining transparency, creating shared motivation, and agreeing on ethical decisions. The discussions around collaborative solutions to the documented challenges are described. The power dynamics at play to negotiate the trade-offs needed to overcome the different constraints are analysed.

#### Using the right methodology based on an appropriate research question constitutes a significant challenge

The research question that the ICRC and the MoPH sought to answer was how to account for the low utilization of sexual and reproductive health services for women attending the ICRC supported facilities. The gap in understanding was a key and common issue discussed between the ICRC and the MoPH PHC teams at field and management levels [1]. While the field response teams (ICRC, MoPH) had a good understanding of the operational context, using the right research methodology to answer this question depended on the precision of the research question, on whether the ICRC and the MoPH could realistically answer it, and whether the academic partner understood the specificities of the context [22]. The FXB team had experience in conducting operational research with humanitarian organizations but lacked in-depth knowledge of the situation of affected populations in Lebanon in terms of existing monitoring records, constraints on unregistered refugees, and geographical as well as cultural and political specificities of the areas selected. In order to build more equal understanding, this gap was overcome through an initial two-week scoping field visit proposed by the academic partner to explore the complex humanitarian response, the nature of the Lebanese health system, the epidemiological context (including the several different populations being served) and the field operational constraints.

In this scoping visit, existing monitoring data were analysed collectively and discussed. Unmet needs in the literature and in the Lebanese context included the lack of preventive services [55–57], high out-of-pocket payments [38, 58] and high use of emergency obstetric care services [38]. The research question was driven by the need to understand who was missed and why. Formulating the research question involved recognizing the relevance of diverse skills, the constructive engagement of field response personnel and the assurance that each partner’s interests would be represented and respected [59–61]. Precise framing of the research question was formulated in the scoping visit through conversations between and among the ICRC, the FXB team in the field, the MoPH, and Skype calls with the FXB team leader in Boston. The scoping visit was essential to discuss constraints, express expectations and start building trust in a joint leadership structure [8, 59]. The results of the scoping visit in specifying the research question also permitted reaching a written agreement over: a) the decision to cross-analyse population-based and facility-based surveys; b) the choice of a sampling frame that would ensure that everyone would be included; c) the use of qualitative interviews to understand why people were missed; d) the decision to build a questionnaire together and e) the dissemination of findings. This agreement in effect empowered the MoPH and ICRC response actors while assuring the FXB partner on the project’s operational feasibility and technical validity. This proposal was formally signed by the three leaders of the research team and served as a statement of commitment to a process of inquiry that guided us all through the next phases of field investigation, data analysis and writing of the report.

#### Failing to account for bias, study limitations, and lack of statistical data are also seen as major shortcomings

In this study the joint teams attempted to resolve the trade-offs between comprehensiveness and feasibility. Together, they analysed and addressed the different possible biases (observer, selection, and those implied by decisions on statistical power). The partners agreed that the main aim was to respond to the core research question in a way that would be operationally relevant, enabling better response to unmet needs within contextual specificities and response capacity. The academic partner entered the collaboration with an interest in many different aspects relating to the registration status of the refugees, their living conditions, and their sense of human security but these questions fell outside the research focus of the response partners on specific issues of refugee and host health needs and health-seeking behaviour. Given the limitations of funding, time and security imperatives, it was agreed by consensus to make the research more strictly operational [5]. To ensure quality and appropriateness of the questions used, the questionnaires were discussed with the health team at ICRC headquarters and the MoPH, translated into Arabic, back translated into English and piloted. The questionnaires were designed using the Qualtrics software package and the data were electronically captured in a secure off-site server for statistical analysis by the academic partner [1]. The team of interviewers, mainly Lebanese Red Cross (LRC) volunteers who were paid a nominal stipend, underwent a 2-day standardized training. This training was administered by FXB Center researchers to minimize interviewer bias and emphasize the importance of respectful data collection, with a particular focus on gender, cultural and historical differences between and among populations studied. To obtain a more in-depth understanding of issues identified at the community level by ICRC and MoPH field teams, a qualitative component was added through Focus Group Discussions (FGDs) with key community members of both Syrian and Lebanese populations. The FGDs were audio recorded with the consent of the participants and, to ensure optimal quality, were transcribed verbatim by native Arabic speakers with medical backgrounds who were familiar with Lebanese and Syrian Arabic. This text was then translated into English by a native English speaker, a researcher from the FXB Center fluent in Arabic.

#### Specifying the population to be studied in conflict affected areas (including populations on the move) involves balancing issues of comprehensiveness and practicality

The difficulty of including unregistered refugees [38, 58] in the sampling frame was overcome through the following methodological strategies discussed with the academic partner:

- To capture health needs from a population perspective, a cross-sectional survey questionnaire was developed to gather data from households living in the catchment areas of ICRC-supported facilities
- To understand the appropriateness of ICRC supported services, a clinic survey questionnaire using a Likert scale was developed
- To include all potential sub-groups in absence of registers, specific aerial Geographic Information System (GIS) mapping tools were used and an experienced ICRC GIS officer, with technical support from the FXB team, built a two-stage cluster-based randomized mapped sample of the target population [1].

By deliberate design and methods of data collection, to make sure that respondents were protected, it was made impossible to retrace the identity or location of any specific person responding to the questionnaire. Respondent confidentiality was further ensured using anonymized and non-linkable data, an ICRC data protection requirement that, after discussion, the academic partner accepted. The FXB Center would have preferred to collect data that included the geolocation variables in order to analyse findings according to socio-economic variables. From the ICRC perspective the risks of collecting, storing and managing GIS data were greater than the benefits to the data analysis and to the beneficiaries. GPS data were not recorded in the tablet, to grant an extra layer of protection of personal data of the participants. This decision required a major negotiated trade-off between protection and precision. In these settlements, where poor Lebanese lived near poor Syrian refugees, and the very poor refugees lived in informal tents, location was proxy for socio-economic status. Without data on location of individual informants, it was impossible to assess their responses in terms of this variable.

#### Measuring the initial health status of displaced populations is difficult especially in conflicts of long duration where essential baseline information is usually missing

Two issues with existing research or data availability were overcome through academic technical advice:

a) Existing sampling frames used for Syrians were based on (or calculated using) United Nations High Commissioner for Refugees (UNHCR) registers or convenience sampling [45, 58, 62, 63]. These approaches potentially (partially or totally) missed an estimated half million unregistered Syrian refugees [38, 58], while the ICRC fundamental intent was to include all [1]. To ensure that all vulnerable Lebanese and registered or unregistered Syrian had a similar probability of being included in the study required the use of GIS sampling.

b) Given the scattered humanitarian engagement in delivery of primary health care [44] despite MoPH continuous efforts [43], a crucial question was whether service gaps identified through facility-based data were covered by other actors or not. Therefore, the central axis of the research was to cross-analyse population-based and clinic-based information to find out if specific groups or expressed health needs were systematically missed.

#### Securing the functional balance of resources (such as financial, technical, human, and time) may prove daunting

In technical terms, conducting the research with existing ICRC and MoPH team members was possible only because of the specific roles, profiles and strong motivation of the participating national and international staff. It was key to engage senior team leaders who had research skills and the authority to adapt the subsequent operational response. The involvement of senior managerial staff in initial steps permitted the opening of a balanced negotiation space for the duration of the project. The background academic and public health training of MoPH and ICRC core team members allowed discussions to take place on common ground. The sound GIS capacity of the ICRC in-country team was essential for the area cluster setup. In parallel, the interaction with the FXB team constituted an opportunity for all the involved staff to learn and acquire research skills -- especially technical capacity in research ethics and field sampling methodologies.

The time trade-offs required by the field research comprised only one aspect of the time challenges baked into a project where the academic team was often in a different time zone. Yet the teams managed to stay involved on a very frequent basis via Skype calls and emails. The time allocated for coordination, planning, and research was in addition to the usual workload of all actors. The time trade-offs for the ICRC field staff were partially compensated by an opportunity to learn, to analyse the existing response through the lens of research, to nurture the understanding of operational complexities and to build research skills. Furthermore, the timing phases of the study had to be adjusted to adapt to the ICRC operational envelope, a balance between what was feasible within the ICRC field budget while remaining acceptable to the academic partner.

#### Adapting methodologies for field conditions becomes troublesome because lengthy prospective cohort studies or randomized controlled trials (RCTs) are difficult to conduct in unpredictable and volatile environments

In order to maintain technical standards for data collection from the field, as recommended by the academic team, a number of difficulties had to be overcome, requiring more time and skills. These issues were met by combining the complementarity of the three partners in terms of knowledge, technical capacities and continuity in key positions.

Using a GIS two stage cluster-based population sample [1] was essential to allow inclusion of all population groups but required specific field visits to inform the sampling process, convey prospective information to key stakeholders in hard to reach areas and conduct adequate trainings to ensure the proper use of geospatial maps. The FXB team’s field presence allowed the researchers to determine the parameters of the GIS sampling while incorporating the difficulty of the terrain (border areas), the complexity of the clustering methodology, and the necessity to make many adjustments in a short time frame.

Choosing to combine population- and clinic-based surveys was necessary in order to understand who was missed and what needs were unmet [1]. This design led to several complexities. First, the design required additional field visits and more manpower to meet the methodological requirements. The FXB team, in the interest of ensuring an unbiased approach to clinic surveys and to support the ICRC field research effort, proposed to have its Arabic-speaking researchers participate in the field research. The ICRC agreed that FXB researchers would augment the ICRC teams and would conduct clinic surveys. Second, this accommodation required the ICRC to engage in further field negotiations to explain why such a complex research design was necessary. Reciprocally, direct participation in the data collection required the FXB team to accommodate to the ICRC’s tight research schedule. Yet the benefits were important: The ICRC field team had the opportunity to consult in real time with the FXB researchers on questions of sampling methods and the FXB team gained deeper understanding of the complex operational, security and administrative regulations enmeshed in the work of both the ICRC and the MoPH.

#### Constraining research efforts are distortions imposed by issues of security and logistics

To allow sufficient time for quality data collection, in each site 16 teams of 2 interviewers each were deployed, each team conducting five 45-min interviews per day over a five-day period, for a total of 400 interviews per site and 1479 households approached [1]. Each team was supported by an ICRC team member onsite everyday resolving logistical constraints and providing guidance on sampling based on geospatial maps. The LRC volunteers relied on their own organizational hierarchy to communicate issues which were then solved between both program coordinators (ICRC, LRC). The scheduling burden on human resources was partially overcome by mobilizing ICRC field teams to include national and international, health and non-health, as well as management personnel. The initiative was an overall team effort and the trade-off was the time diverted from field operations resulting in delays in routine activities. Conversely, this extra mile was supported by ICRC management because of the expected value of achieving a better understanding of operational issues to be addressed in subsequent planning. The effort also represented an opportunity to engage in remote areas and build team relationships among senior MoPH, ICRC and FXB staff — critical to the continuity of the study between 2016 and 2019.

While the ICRC team was relying on the FXB for technical input on methodology and analysis tools, the academic team was relying on ICRC and MoPH field staff for managing all aspects of field preparation and security access. Consequently, the joint pace of field implementation was slower than initially planned. The ICRC, based on transparency, engaged with different stakeholders including municipalities, security forces and influential community groups or leaders to explain the purpose of the study and ensure a smooth process. The FXB team also had to rely entirely on internal processes for field security and abide by ICRC and MoPH operational rules.

#### Unstable and unpredictable funding patterns restrain the perceived scope of research

The financial barrier was partially overcome by integrating the resources for the research into the regular ICRC 2016 and 2017 field program at an affordable rate. The direct costs of the research included only FXB team field visits. The budget did not cover additional time allocated by the FXB, ICRC or MoPH teams for off-site work or regular in-depth Skype discussions to resolve issues. Financially, the direct costs of the study represented around 10% of ICRC PHC direct costs for the program, with a potential critical return on investment in terms of refining the appropriateness of care delivered. The uncertainty in estimating the exact budget and duration of the study required mutual trust, the support of the ICRC Beirut operational management team, and an overall capacity of ICRC field actors to be flexible for budget management issues. The indirect economic costs of ICRC, MoPH and FXB contributions to this research effort, in terms of human resources and time, were not included and represent an important “sunk cost” for each partner, which each absorbed internally. The indirect costs for the ICRC involved putting an additional burden on busy teams: the expected added value was the knowledge gained to help guide future response. Another issue involved the status of the academic partner, the ICRC headquarters, and different contractual obligations. The timing requirements at the field level in Lebanon demanded a rapid start. Hence the FXB Center relied on a flexible consultancy process, negotiated at the level of the ICRC Beirut delegation. The work could thus be conducted within the operational framework of the organization, which created real clarity and stability for the technical partner. This arrangement permitted essential operational latitude and speed but left the ICRC Geneva headquarters (HQ) distanced from the process, resulting in the need to include the HQ health team on decisions and findings in an ex post facto mode.

#### Ethical issues are complex and in certain situations of marked power differentials can appear prohibitive

ICRC facility-based information for monitoring purposes relies on existing aggregated anonymized processes, collecting personal data exclusively within the ICRC data protection frame [64]. As this study included individual and household visits and interviews, an additional ethical review was necessary, managed by the academic partner, welcomed by the ICRC and approved by the MoPH. The ethical approval was sought from the Institutional Review Board (IRB) of the Harvard T. H. Chan School of Public Health, which was granted upon submission of the study protocol and conditional on the ethical approval of the MoPH which was received. All research staff had to adhere to the ICRC code of conduct. No internal or external participant who had not received the ethical training from the FXB Center researchers was allowed to join the interviewers’ teams.

Discussing and acceding to these ethical requirements created a strong sense of purpose and cohesion among all members of the combined research teams. Adherence to the ICRC code of conduct required the FXB team to acknowledge the field dynamics--and only then made it possible for them to have access to the population under study. These agreements allowed each leader to manage protection, confidentiality, ethical and contextual issues within and among respective teams. The process by which FXB and ICRC staff adapted to joint field-group dynamics based on shared expertise, equality of status, respect and interdependence was in the main a mutually enriching experience for both teams. Despite careful oversight and agreements, however, one early situation of relational tensions had to be monitored, discussed and managed accordingly.

Given the time constraints of the ICRC and the MoPH and the prospect of a lengthy IRB process entailed in attempting to obtain ethical approval to interview adolescents below age 18, it was decided that adult caretakers of these younger adolescents would be sought to represent this specifically vulnerable group. The recognized trade-off in this decision was that the researchers could not capture the independent views of this younger population.

The research was conducted respecting official working hours and religious celebrations during which the field work was stopped. Whenever people interviewed needed medical care, they were referred in accordance with standard operating procedures of the ICRC and the MoPH [5, 33]. To grant priority to the health needs of a population under study is also a prerequisite of gaining academic IRB approval but usually operationalizing this requirement requires considerable advance planning and negotiation with local actors. It was a significant boon, from the academic perspective, that this aspect of the field research could rely on prior pathways of referral and care.

In terms of benefits to the general population affected, the preliminary results of the study were used as the basis for substantial recommendations to re-orient the response then ongoing in the field [5, 8, 33]. There was a shared ethical commitment to “do no harm” to protect the response capacity of local actors beyond the time of the research [33]. This responsibility included anticipating and minimizing potential negative side-effects of the study on beneficiaries and on protecting the overall acceptance of the ICRC and MoPH among the communities. One positive side-effect of the cross-sectional population-based questionnaire was to raise awareness among all community members of ICRC supported services.

#### The differences in the analytic cultures of humanitarian as compared to academic actors constitute yet another type of barrier

For the academic partner, the tenth challenge surfaced at the beginning and the end of this collaborative research journey. It was embedded in the initial decisions not to maintain records of the geographic coordinates of those interviewed (thus making it impossible to correlate findings with location) and not to seek retrospectively reasons for participant refusals to participate. In these instances, the priority was placed on finding the information that would be of use to the operational actors and in adhering to ICRC codes of conduct regarding protection of individuals. The academic partner determined that these compromises were acceptable in order to gain further insights into the dilemmas and challenges of humanitarian action in general and in the context of Lebanon.

This tenth challenge also arose very early in discussions that defined the grounds for participation of an academic centre in the research endeavour. Traditionally universities demand sole authority on copyright and access to data gathered during the study. Fortunately, from prior efforts and experience in humanitarian settings, the FXB partner had negotiated with the university that copyright and access to data sets might be joint. This prior set of efforts allowed the FXB team to enter at the beginning of this collaborative investigative effort with an openness to the range of modes of publication.

For the humanitarian response actors, the capacity to create a learning space to integrate analytic information from the field is challenging in a competitive humanitarian arena where the focus is on obtaining quick positive results [21]. Discussing and sharing the results at different levels, including negative results, proved critical to adapting the subsequent health response and interpreting the identified gaps as an opportunity to change rather than a sign of failure. This partnership contributed to developing a “culture of enquiry” [24] among field responders and managers to empower them to discuss practical solutions [6, 24].

The results of the study suggested that there was a mismatch between the services supported and the expressed needs at population level [1]. The study also showed a lack of community awareness of these services [1]. These results could not have been inferred from monitoring data at the facility level only. In order to share learnings and decrease power differentials related to academic knowledge, the ICRC staff engaged in the research and the academic partner developed a joint diffusion strategy explaining the power of the combined population- and clinic- based surveys. Progress updates were presented regularly by field teams including FXB members to key ICRC Beirut health and management staff affected by the findings. At the conclusion of the field research, preliminary results were presented by senior FXB and ICRC team members at the Geneva HQ and Beirut Delegation levels. Early discussion of the results allowed the ICRC Beirut health team to explain the operational implications prior to the completion of the formal internal ICRC comprehensive report.

## Discussion

This experience allows us to describe how the models of academic- or humanitarian- driven inquiry were accommodated in a joint response to a documented research gap. We also explore how this partnership allowed us to go beyond some of the limitations observed in the literature.

### How the FXB center went beyond academic driven research

What drove the immediate recognition that the FXB team would be the technical partner rather than lead the endeavour were factors of experience in other refugee settings and philosophy of approach. The FXB research team knew that when an effective government controlled access to the refugee populations and all health interactions were conducted through field actors, the role of academic researchers would need to be to complement these other factors and competencies. Access to the population would be mediated by those responsible for security and the research questions would have to be of fundamental operational interest. The FXB team also knew that the extent to which issues such as appropriateness of care or observance of norms of human rights would be discussed depended on the volatility of the situation and the integrity of the humanitarian partner. The FXB team’s prior knowledge of and respect for the MoPH and the ICRC shaped the team’s confidence in entering the work as a technical partner, knowing that the ultimate result would be of benefit to all parties, including the beneficiaries. The value of the investigation, including the different insights gained in the process, was determined by the FXB team to be more than worth the gap in funding that was not accounted for in the relatively modest envelope integrated in the Delegation budget.

Furthermore, all FXB team members knew that the partnership would be intellectually fruitful and engaging. Key members of all partners shared a high regard for the power of epidemiological inquiry and recognized that structured quantitative inquiry could yield important understandings for operations in even the most distressed settings. These shared values and skills provided a crucial bedrock on which to build the collaboration.

The FXB researchers understood in general the issues facing refugees forcibly displaced in war but the particularities of their circumstances as self-settled populations in poor communities in Lebanon were of significant specific interest to the team. In this vein, the FXB knew from past experience that such work would require time that would not be compensated. The team also anticipated that what would be learned, generally and for the academic community, would be of great value. The unequivocal requirement for sufficient time is described in global health and academic partnerships [18, 23, 26] and is essential if academic researchers are to engage in humanitarian settings.

The FXB and ICRC teams recognized from the outset that the issues of conflicts of interest, ownership of data, and right to publish would require in-depth discussion, keeping in mind that these issues might potentially mean that operational constraints could override a publication agenda, should the protection of or access to the affected population be at stake. The fact that lead researchers from the ICRC and MoPH were academically trained combined with the readiness of the ICRC as an institution to explore the research question made the discussion very straightforward and allowed FXB researchers to focus on supporting the ICRC’s field research.

The need to be able to move away from the prime objective of publishing results to improving the operational response was one key pillar of the discussion. Another was mutual acknowledgment of the need to discuss the interests of each partner in the use of results and the presentation of the results in modes that would support the beneficiaries.

### How the MoPH and the ICRC went beyond humanitarian-driven approaches

The research question was driven by observations of the response actors (ICRC, MoPH) who wanted to understand why so few women were coming to the supported services and the academic partner was brought in to help answer that question. For the ICRC and for the MoPH, the decision to divert resources from responding to the needs of beneficiaries to conducting research was a necessary and expected challenge [24, 32]. Research can still be perceived by humanitarians as less operational when compared to field response, relating to the difficulty of producing relevant recommendations rapidly enough [5, 22]. Longer term integrated and flexible funding was essential -- often not the case with humanitarian funding cycles even in protracted crises.

The need to be transparent in a world competing for short planning and funding cycles also had to be part of the journey, especially when unexpected results challenged the internal capacity to learn from the research process [18]. For example, the finding that supported services were not fully utilized and that the program design did not meet the key health complaints of the population had to be transmitted and absorbed in a positive mode in order to reorient the response and feed into subsequent policy discussions [1].

Furthermore, continuity of key MoPH and ICRC staff was crucial but proved challenging in the context of rapid turnover -- a struggle identified in much humanitarian and global health research [18, 22, 28, 32]. Continuity of engagement in the research ensured coherence, which is essential in building individual learning capacity [18, 26]. Staffing changes can result in minimal uptake of findings, missed opportunities, institutional memory loss, and little return on investment [18, 22]. When human resources are a challenge, mutual cross-task learning among team members is important and capacity building at individual levels is essential to produce sustained results [26]. Central to this partnership was the constitution of a stable core team including both ICRC and MoPH junior and senior staff.

Finally, the customized use of GIS techniques for specifying the sampling frame, the academic partner’s experience in complex sampling in conflict settings as well as updates on recent research initiatives in the region opened a collective thinking and learning space [18], an enriching experience for the humanitarian staff. The debates over methods, processes, and results challenged the field staff and constructively changed their perspective on the research endeavour. The humanitarian staff need to develop these understandings and welcome the academic analysis that situates findings in a wider realm of intellectual debate. Although unsettling for some staff members, the task of writing a publication, from use of rigorous methodologies to referencing the scientific literature, allowed the humanitarian team to expand their view of their own work and the efforts of the many others devoted to humanitarian response [1].

One important specific challenge for the MoPH was to undertake to seek the opinion of the community served in a context where the expressed issues might be very difficult to respond to in practical terms. To be able to deal with such concerns as trust in the public health system, for instance, would require addressing many broad historic and structural determinants. In addition, the research was conducted in areas with security situations which truncated time for follow-up -- leading to unanswered questions for the MoPH or prompting an interest in further in-depth investigation. Finally, even if it were clear to the MoPH that the community’s concerns would best be met by enacting universal health coverage through primary health care, the overwhelming question in the current Lebanese context is feasibility [50]. Yet although broad structural issues were difficult to address, the partnership confirmed the urgent need to raise awareness about the availability of good quality services, continue the expansion and improvement of services, and accelerate outreach within the population.

## Conclusion

The ten identified challenges were all present in the collaborative research effort described here. To meet these challenges required varying degrees of compromise and adaptation for each step. Reflecting on this experience of a small and motivated research team working on this relatively modest initiative permits specification and discussion that may be of potential use to those embarking on similar partnerships in the future [59–61].

Abundant work went into the front end of this collaborative effort. Identifying a research issue of joint interest depended on the individual and collective capacity to share expertise and allocate time for preparedness. Considerable effort had been expended to understand the health issues of the population, data availability and gaps in knowledge. The ground had been defined, in effect, in such a way that virtually called for the kind of inquiry the research team embarked upon. Fostering the involvement of a diverse group of actors (academics, humanitarians and public health authorities) at the earliest stage created a shared readiness to construct a multi-faceted understanding of the issues and their inter-relationships.

Methodological challenges proved relatively easy to negotiate because the team was small, and the leaders had a sense of shared expertise and interdependence. Building a stable multi-skilled team allows all partners to mobilize their research potential. The different capacities of different actors must also strongly align with a respect for the strength of epidemiologic methods. Relying on these methods-- appropriately adapted for statistical power to obtain information in varying field settings on the health needs of diverse populations—meant that all actors learned how to discern and elucidate the crucial factors and relationships that undergird the provision of relevant services to populations in war and displacement. The research effort involved paying focused attention to teaching, de-briefing, and discussing a myriad of findings with many senior and junior participants in the research endeavour—those in the field and those who worked more from the delegation office in Beirut. In such ways, it was possible for the core research group to embed institutional learning useful to future efforts to understand key issues by relying at least in part on empirical field-based findings.

Creating and maintaining a participative mechanism for decision-making and transparent space for negotiation are delicate processes. The main challenges in this research project lay in the ones identified in the literature regarding the management of issues relating to security and logistics, ethics and norms, and to organisational cultures. While the FXB team appreciated the streamlined aspects of this project to the extent that the ICRC handled all the tricky and subtle aspects of security and logistics, this arrangement introduced a distance from the daily negotiations and created for FXB an unaccustomed sense of disconnection from the field dynamics. Mutual respect for specific spheres of decision-making was essential to re-setting the equilibrium and sustaining the partnership. Creating this channel of discussion begins with early acknowledgment and recognition of the different competencies of each partner. Mutual trust sustained throughout the partnership helped to account for power inequalities in particular spheres of command or expertise and permitted sharing of uncertainties. Issues of research ethics and power differentials arose during this collaboration. Because questions related to geospatial localisation were not permitted, substantial information was consequently not obtained. But this loss, weighed against the anticipated robust results, was resolved through respectful argument. The organizational identities and responsibilities also need to support the choice of modalities for dissemination of results (publication vs. internal reports) within a more global longer-term perspective that seeks to include the needs and requirements of all partners and builds upon their respective strengths.

The broader institutional trust and autonomy of the core research team further supported resolution of issues during the intense pace of calls that kept the communication channels clear and open. Problems could be handled in very real time over 2 years. The choice of the senior actors to maintain overall flexibility and field management permitted a temporary relative release from institutional processes. To reduce some of the complexities of collaboration between a field-based organization and an academic institution, it is suggested that relative operational and financial autonomy should be designed into the management of the research. We also recognize the importance of having funds embedded in existing institutional mechanisms in order to be able to secure the essential link between empirical research outcomes and influence on the subsequent planning phases of the response.

### What academics should keep in mind

Time is needed to develop a trusting relationship, to define the relevant research questions and select the appropriate methodologies collectively. The research design should become part of the operational response, grounded in the operational reality. The research efforts in the humanitarian sphere should constitute an interactive part of the operational response, where the previous extensive field knowledge of humanitarian contexts by academic team members is key, so that research questions and results can be used for improved operational and policy response. The academic skills developed both by ICRC and MoPH staff prior to this joint effort facilitated in-depth technical discussions, allowing an active engagement in prioritizing key issues and selecting appropriate research tools, such as the GIS mapping. Academic partners should develop the ability to engage on equal footing with humanitarian and health authority actors in order to provide rapid preliminary results useful for important operational decisions, to nurture the operational thinking with updates from the broader literature, and to receive feedback on early results.

### What humanitarians should keep in mind

Embedding operational research in humanitarian operations will be done either to the detriment of the research or the operations unless there is a pre-defined time and commitment of financial resources to support all partners in the research. Allocated resources are needed to allow each team member to contribute to the discussion through the lens of his or her specific competencies and to engage in challenging discussions, always tethered to the need to solve operational issues. Adequate time to work together should be factored into the routine work of health authorities and humanitarian actors if such joint initiatives are meant to be sustained. Time is needed for joint adaptations, for creating a shared vision, for securing continued funding and for anticipating the next phases of research work.

Field partners should allow the academic thinking and analytical process to take place, involving field personnel as the results take shape. Collaborative processes with academic partners can accelerate integration of research findings into the operational and policy reality, linking early results with planning processes. Allowing key staff engaged to be part of the research process irrespective of their field assignment, which allows a longer personal learning process and perspective, can be very helpful. The link to an updated set of broader academic literature, writing skills and technical tools is difficult to maintain in the humanitarian setup and can be nurtured and developed together with academic partners.

Joint research involving field actors and academics has the potential to contribute to improved responses for the most vulnerable affected by complex protracted crisis if it is conducted with proper resources, mutual respect for competencies and constraints, and trust in a shared vision. As the challenges of sustaining effective humanitarian operations in conflict settings increase, it is only prudent to consider how to marshal the resources of research partnerships to help define these challenges and suggest operational interventions to make the humanitarian response tighter, more equitable, and ultimately more effective.

## Data Availability

The data that support the findings of the primary field study and the debate are available from the ICRC but restrictions apply to the availability of these data due to confidentiality of information, and so are not publicly available. Data are however available from the authors upon reasonable request and with permission of the ICRC.

## Abbreviations

EWEC: Every Woman and Every Child Everywhere
FGD: Focus Group Discussions
FXB: François-Xavier Bagnoud
GIS: Geographic Information System
GPS: Global Positioning System
HQ: Headquarters
ICRC: International Committee of the Red Cross
IDPs: Internally Displaced Populations
IRB: Institutional Review Board
LRC: Lebanese Red Cross
MoPH: Ministry of Public Health
MoSA: Ministry of Social Affairs
MSF: Médecins Sans Frontières
NGOs: Non-Governmental Organizations
RCTs: Randomized Controlled Trials
SORT-IT: Structured Operational Research and Training Initiative
SRH: Sexual and Reproductive Health
UNHCR: United Nations High Commissioner for Refugees
